# Trapped DNA fragments in marine sponge specimens unveil North Atlantic deep-sea fish diversity

**DOI:** 10.1098/rspb.2023.0771

**Published:** 2023-08-30

**Authors:** Erika F. Neave, Wang Cai, Maria Belén Arias, Lynsey R. Harper, Ana Riesgo, Stefano Mariani

**Affiliations:** ^1^ School of Biological & Environmental Sciences, Liverpool John Moores University, Byrom St, Liverpool L3 3AF, UK; ^2^ Natural History Museum, Cromwell Rd, South Kensington, London SW7 5BD, UK; ^3^ The Freshwater Biological Association, The Hedley Wing, YMCA North Campus, Lakeside, Newby Bridge, Cumbria LA12 8BD, UK; ^4^ Department of Biodiversity and Evolutionary Biology, Museo Nacional de Ciencias Naturales de Madrid, Calle José Gutiérrez Abascal 2, 28006 Madrid, Spain

**Keywords:** environmental DNA, biodiversity, marine monitoring, sponges, metabarcoding, extended specimen

## Abstract

Sponges pump water to filter feed and for diffusive oxygen uptake. In doing so, trace DNA fragments from a multitude of organisms living around them are trapped in their tissues. Here we show that the environmental DNA retrieved from archived marine sponge specimens can reconstruct the fish communities at the place of sampling and discriminate North Atlantic assemblages according to biogeographic region (from Western Greenland to Svalbard), depth habitat (80–1600 m), and even the level of protection in place. Given the cost associated with ocean biodiversity surveys, we argue that targeted and opportunistic sponge samples – as well as the specimens already stored in museums and other research collections – represent an invaluable trove of biodiversity information that can significantly extend the reach of ocean monitoring.

## 1. Introduction

The worrying and widespread trend of ocean biodiversity loss that typifies the Anthropocene calls for increasingly powerful and accurate approaches to expose the nuances of this loss, understand its main drivers and inform mitigation strategies. One such recent scientific advance has been ‘environmental DNA’ (eDNA) analysis, an approach by which collecting DNA fragments shed by organisms in their habitat allows researchers to generate biodiversity data at unprecedented scales [1] and granularity [2], redefining the way we observe and understand ocean life.

Biological research collections are critical for eDNA analyses. Apart from expanding DNA taxonomic reference databases from tissues [3], they also provide untapped genomic insights that have become more accessible with the advancement of molecular techniques [4]. Metabarcoding, in particular, allows for ecological insights, such as detecting multi-decadal community shifts from eDNA in ethanol-preserved ichthyoplankton samples [5], or tracking micro-evolutionary changes in the gut microbiome of 100-year-old fish specimens [6]. These are prime applications of the extended specimen concept [7], that is, a comprehensive approach to biodiversity collections that extends beyond the physical object to multiple other uses made possible by efforts such as digitization, and new attitudes towards phenotypic description such as considering holobionts [8,9].

Filter-feeding marine sponges (phylum: Porifera) were recently found to act as natural eDNA samplers, able to retain eDNA fragments reflective of their surrounding biological communities [10]. Sponges are ideal extended specimens, in that exploring beyond the host DNA provides an understanding of the environment from which the sponge was collected. Experimental studies subsequently found that sponge species differ in their ability to retain eDNA, with some species likely to trap DNA for longer intervals than what is usually observed in water samples [11,12]. Given the urgent need to measure trajectories of biodiversity changes, we explored whether this sponge natural sampler approach could characterize fish assemblages across the North Atlantic by leveraging sponge specimens previously collected for other scientific purposes from vulnerable and underexplored deep-sea habitats.

## 2. Results

**Figure 1.**
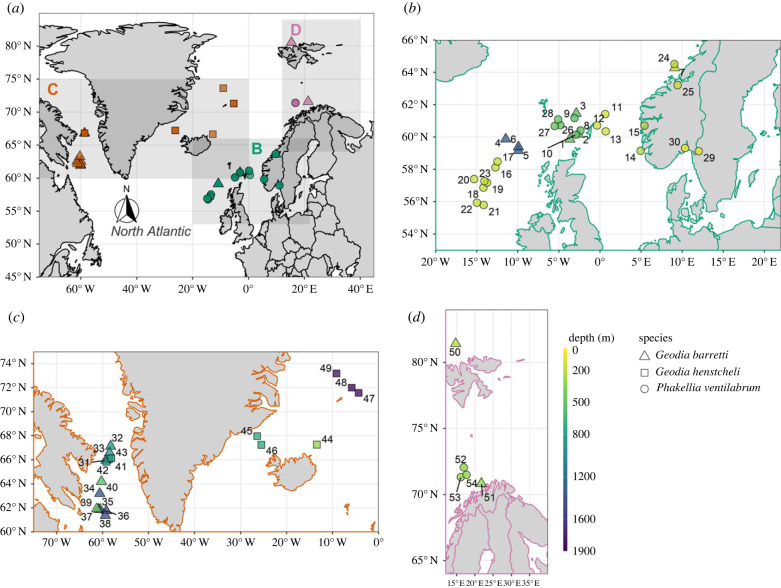
Maps showing locations of sponge specimen retrieval. Depth is indicated by the colour bar and sponge species is indicated by the shape of the points. (*a*) Map of the North Atlantic study area. (*b*) Map of the Northeast Atlantic region. (*c*) Map of the North American Boreal Atlantic region. (*d*) Map of the Norwegian-Arctic Seas Atlantic region. Sponge specimens in (*b*–*d*) are jittered for visibility and labelled 1–30 (Northeast Atlantic), 31–49 (North American Boreal) and 50–54 (Norwegian-Arctic Seas).

We detected natural sampler DNA (nsDNA) from three sponge species (*Geodia barretti, Geodia hentscheli* and *Phakellia ventilabrum*) (*n* = 54, retained from 64 samples sequenced—see Methods) across varied benthic habitats in the North Atlantic (figure 1). The specimens were between 3 and 10 years old, spanning the continental shelf down to the bathyal slope (approx. 80–1900 m), and cover large biogeographic regions such as the Northeast Atlantic, North American Boreal, and Norwegian-Arctic Seas (figure 1*b–d*) [13] (electronic supplementary material, table S1). We amplified a fish-specific 12S mitochondrial rRNA marker (tele02) [14] from the previously extracted total DNA of the sponge specimens, and sequenced the targeted amplicons on an Illumina iSeq 100, resulting in 5,269,740 raw reads. After quality filtering (see Methods), we retained 4 565 067 reads for downstream analyses (electronic supplementary material, table S2), resulting in a median of 12 992 reads per sample (*n* = 74) (electronic supplementary material, figure S1), including controls (*n* = 10) and samples that were later removed (*n* = 10) for having low reads (mostly *G. hentscheli*).

### (a) Vertebrate biodiversity

After bioinformatic processing, the sponges yielded 142 eukaryote molecular operational taxonomic units (MOTUs), resulting in 125 non-human, contaminant-free, marine MOTUs, which could be identified confidently to at least the taxonomic rank of class. Among these, we detected 119 fish MOTUs of which 65 were identified to species level at ≥99% identity to reference sequences, excluding contaminants (electronic supplementary material, tables S2 and S3). The following species were removed from downstream analysis: our positive control (the tropical freshwater catfish *Pangasianodon hypophthalmus*), two species (ie. *Amphiprion ocellaris*, *Pomacanthus imperator*) from a different project processed at a similar time [12], and one Indo-Pacific fish heavily traded as seafood (*Nemipterus zysron*). The fish MOTUs, spread over the classes Actinopterygii and Chondrichthyes, comprised 28 orders, 54 families and 94 genera. A sand sea star (*Astropecten irregularis*) common in deep sea benthos was also detected, while sponge DNA was never detected and likely not amplified, due to their phylogenetic distance from vertebrates. We also removed domestic animals (e.g. *Sus scrofa*, *Bos taurus*) and terrestrial mammals such as caribou (*Rangifer tarandus*), native to the Northern Hemisphere, whose putatively leached DNA was found in a *G. barretti* specimen from the Davis straight, west of Greenland. After these removals, we detected five ‘bonus’ non-fish vertebrate species, comprising three marine mammals (harbour porpoise *Phocoena phocoena*, Atlantic white-sided dolphin *Lagenorhynchus acutus* and Bryde's whale *Balaenoptera brydei*, detected in both the west and east North Atlantic) and two seabirds (pelagic cormorant *Phalacrocorax pelagicus* and glaucous gull *Larus hyperboreus*).

### (b) Biogeography and depth-associated fish assemblages

**Figure 2.**
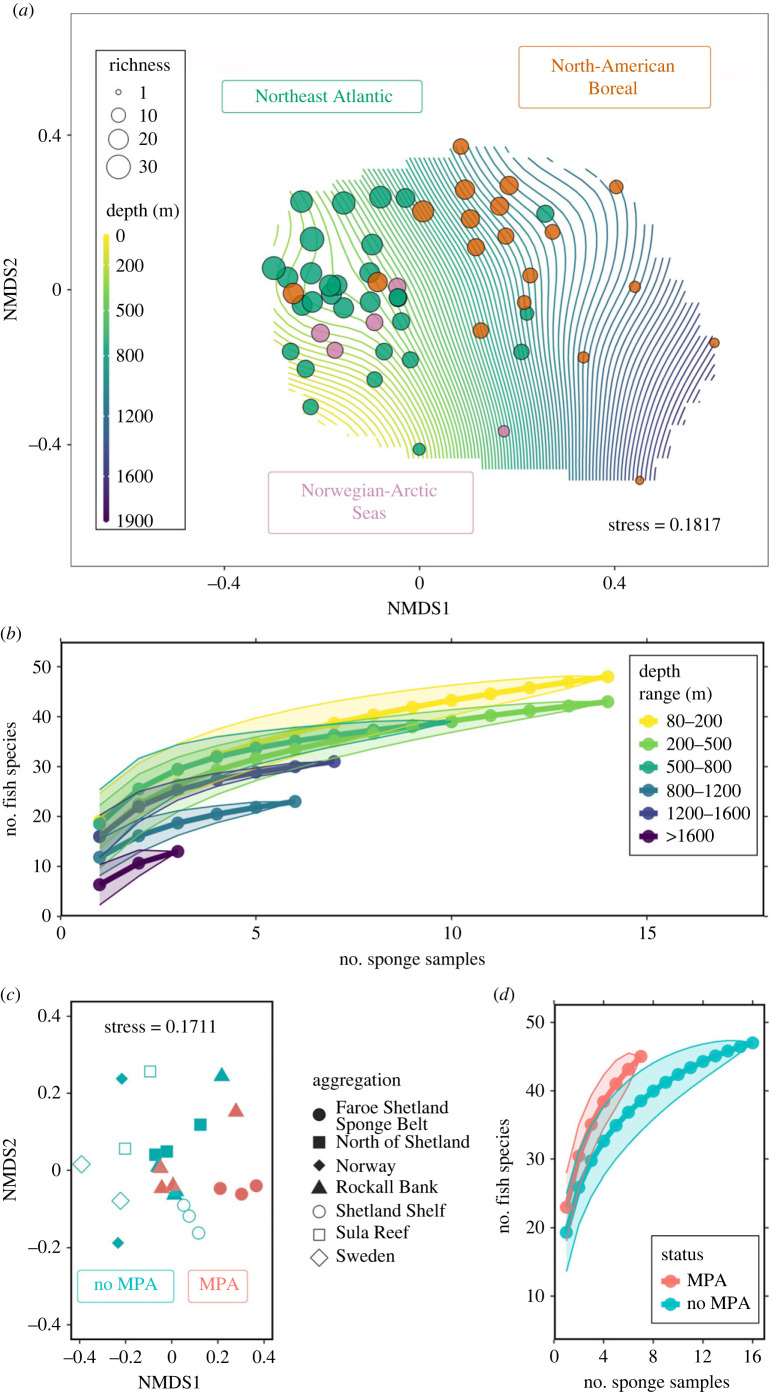
Plots conveying alpha and beta diversity from species-level teleost and elasmobranch detections. (*a*) Non-metric multi-dimensional scaling (NMDS) plot of a Jaccard dissimilarity species matrix, where points are coloured by North Atlantic region and size indicates species richness. Depth is plotted as a surface, where each line denotes a 20 m interval. (*b*) Fish species accumulation curve, grouped by depth range. (*c*) NMDS plot of a Jaccard dissimilarity species matrix of *P. ventilabrum* samples from the Northeast Atlantic region. Points are coloured by MPA status and shapes represent different sponge aggregations. (*d*) Fish species accumulation curve of the *P. ventilabrum* samples from the Northeast Atlantic region, grouped by MPA status.

Fish communities significantly differed between biogeographic regions of the North Atlantic (*R*^2^ = 0.16, *p* < 0.001; figure 2*a*; electronic supplementary material, table S4). Beta diversity was examined through non-metric multi-dimensional scaling (NMDS) of a Jaccard dissimilarity matrix based on teleosts and elasmobranchs detected across sponge samples (consisting of only MOTUs identified to the species level, though the same pattern was observed when including genus level detections, electronic supplementary material, figure S2), and by permutational multivariate analysis of variance (PERMANOVA) testing. The effects of region and sponge species, as well as the potential interactive effect between these two factors, on fish communities were also tested by PERMANOVA, but the dispersions of each group of sponge samples were not homogeneous which PERMANOVA testing is sensitive to. Bearing in mind this caveat, the effect of sponge species was significant (*R*^2^ = 0.08, *p* < 0.05) and independent from the effect of biogeographic regions (i.e. the interaction was not significant), but the regional effect explained more of the variation in fish community composition than the effect of sponge species (*R*^2^ = 0.16, *R*^2^ = 0.08, respectively) (electronic supplementary material, table S4).

Sponge samples appeared broadly grouped into the biogeographic regions previously determined from global distribution data of marine taxa [13], emphasizing the effectiveness of sponge nsDNA to capably distinguish between marine realms (figure 2*a*). Pairwise comparisons of β-diversity revealed that all regions significantly differed, with the North American Boreal region showing greater divergence from both the Northeast Atlantic (*R*^2^ = 0.14, *p* < 0.001) and the Norwegian-Arctic Seas (*R*^2^ = 0.13, *p* < 0.001), compared to the divergence observed between the regions located in the eastern North Atlantic (*R*^2^ = 0.06, *p* = 0.025) (electronic supplementary material, table S4).

Latitude, depth and sampling year were all significant correlates of fish β-diversity. Depth had the strongest correlation (*R*^2^ = 0.58, *p* < 0.001) followed by latitude (*R*^2^ = 0.35, *p* < 0.001) and year (*R*^2^ = 0.17, *p* = 0.018) (electronic supplementary material, table S4). We attribute the weaker correlation with sampling year to be a by-product of the different regions being sampled in separate months. Depth was plotted as a smooth surface over the NMDS ordination plane (figure 2*a*), particularly highlighting how the composition of the Northeast Atlantic sites correspond with shallower continental shelf depths, while the North American Boreal samples follow the gradient of the slope into bathypelagic depths. Species richness approached saturation among all depth ranges from which sponges were sampled, with the least variance in the 1200–1600 m range where the standard deviation decreased with relatively less samples. Fish species richness progressively decreased with depth, except between 1200–1600 m depth, which had a higher richness than the 800–1200 m samples, but also approached saturation most robustly. (figure 2*b*).

To further test the extent to which sponge nsDNA data could be used to distinguish between more fine-scale fish assemblages, the *P. ventilabrum* samples from the Northeast Atlantic were analysed as a subset (*n* = 23) to compare similar habitats and to control for any possible bias introduced by using different sponge species. We observed variance across samples collected in areas with differing levels of marine protection. Species richness appeared to be higher in marine protected area (MPA) sites, and communities detected in MPAs significantly differed from those outside MPAs (*R*^2^ = 0.09, *p* = 0.026) (figure 2*c*,*d*). The same subset of sponges was also tested for significant differences in teleost and elasmobranch β-diversity between various *P. ventilabrum* aggregations (figure 2*c*); however, none of the pairwise comparisons among aggregations were significant after correcting the p-values for multiple testing (electronic supplementary material, table S5). This was likely due to low replication within each of the several locations (e.g. Sula reef, Shetland Shelf) being compared.

### (c) Fish detections and indicator species analysis

**Figure 3.**
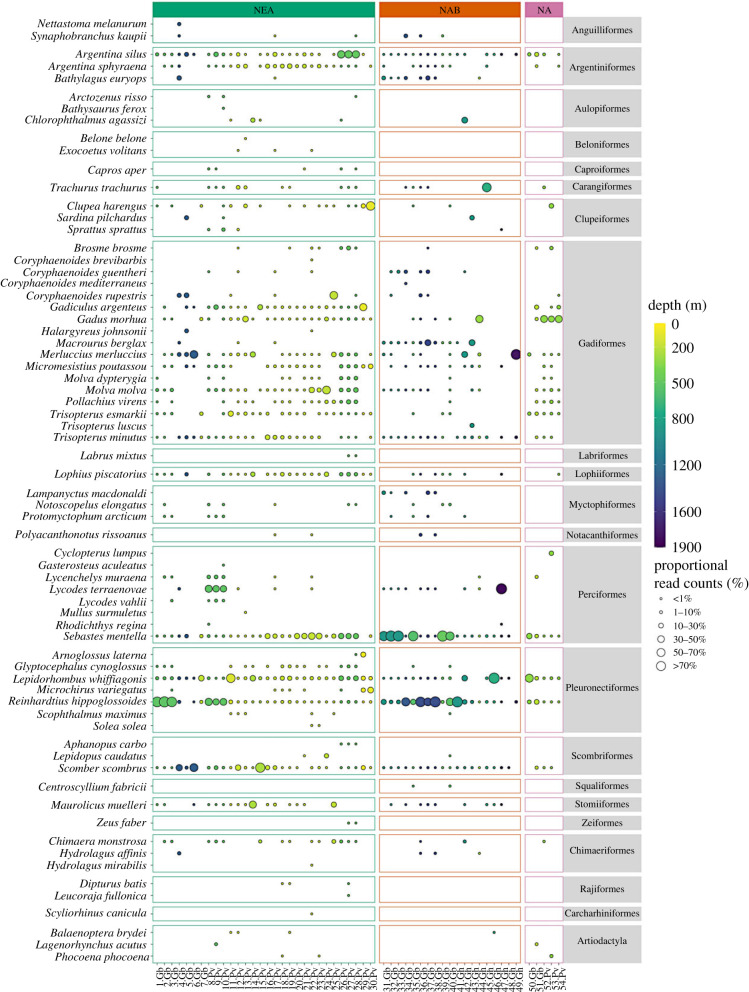
Bubble plot showing teleost, elasmobranch and mammal species detected, where the size of the bubble indicates the proportional read counts of that species represented in a sample. Samples are listed at the bottom, where the number refers to the labels in figure 1 and the abbreviations refer to the sponge species (Gb = *Geodia barretti*, Gh = *Geodia hentscheli*, and Pv = *Phakellia ventilabrum*). The bubbles are coloured by the depth at which the sponge specimen was sampled. The vertical panels separate the sponge specimens by the three biogeographic regions: NEA = Northeast Atlantic, NAB = North American Boreal, and NA = Norwegian-Arctic Seas.

Greenland halibut (*Reinhardtius hippoglossoides*), beaked redfish (*Sebastes mentella*), and megrim (*Lepidorhombus whiffiagonis*) were detected in almost all 54 samples (i.e. 52, 51 and 50 samples, respectively) (figure 3, electronic supplementary material, table S3). Other frequently detected species included Atlantic mackerel (*Scomber scombrus*), greater argentine (*Argentina silus*) and poor cod (*Trisopterus minutus*) (i.e. 48, 46 and 44 samples, respectively), all of which are known to be abundant organisms in pelagic and demersal habitats of the North Atlantic.

While the 12S marker was designed to pick up teleost fish, six cartilaginous fish (class: Chondrichthyes) were also detected. Three chimaeras, the closest living relatives to sharks and rays, were detected, including the rabbit fish (*Chimaera monstrosa*), which was detected in 17 samples. Two elasmobranchs were from the family Rajidae: the shagreen ray (*Leucoraja fullonica*), which is IUCN red-listed as vulnerable, and the blue skate (*Dipturus batis*), which is critically endangered (both detected in the Northeast Atlantic; figure 3).

**Figure 4.**
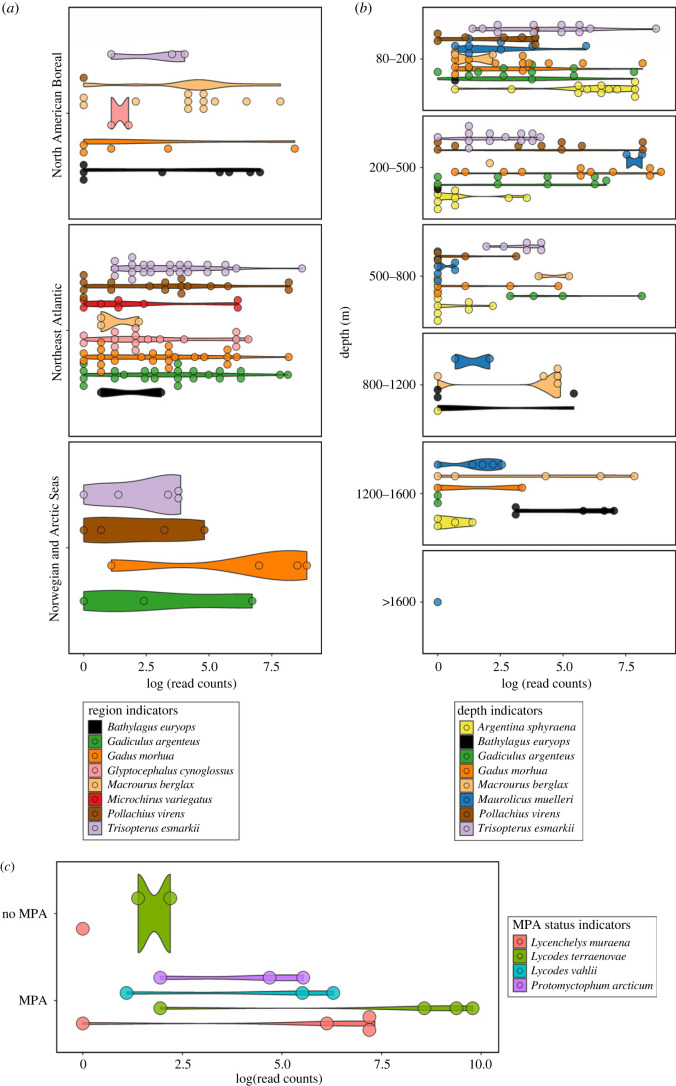
Violin dot plots of log-transformed read counts, highlighting identified indicator species. (*a*) Violin dot plot of indicator species associated with biogeographic regions. (*b*) Violin dot plot of the top eight indicator species associated with depth. (*c*) Violin dot plot of indicator species associated with MPA status.

Indicator value species analysis conducted across biogeographic regions and depth ranges (figure 4*a*,*b*) detected eight species as biogeographic indicators, and 16 species as depth layer indicators, with seven species identified as indicators for both region and depth (electronic supplementary material, table S6). Indicator values (A, B, stat) were calculated using presence–absence data to conservatively interpret detections. ‘A’ is the estimate probability that samples are associated with a region or depth layer if the indicator species has been detected in the sample (i.e. specificity or predictive value). ‘B’ is the estimate probability of detecting the indicator species in a region or depth layer (i.e. sensitivity). ‘Stat’ is the indicator value index, which suggests the strength of the indicator species association and encompasses both ‘A’ and ‘B’ values.

Many species of commercial value had strong significant associations for both region and depth range. Norway pout (*Trisopterus esmarkii*) was positively associated with the Northeast Atlantic and Norwegian-Arctic Seas (stat = 0.857, *p* < 0.0001) (figure 4*a*) and had a strong association with depths ranging from 80–800 m (stat = 0.903, *p* < 0.0001), such that there was high specificity (A = 1) or likelihood that a Norway pout detection occurred in habitats shallower than 800 m depth (figure 4*b*, electronic supplementary material, table S6). Saithe (*Pollachius virens*) shared the same region and depth associations as Norway pout, although to a lesser strength. Atlantic cod (*Gadus morhua*) also showed clear associations with the eastern Atlantic between 80 and 800 m (figure 4*a*,*b*). Roughhead grenadier (*Macrourus berglax*) and blacksmelt (*Bathylagus euroyops*) had a strong association with the North American Boreal with grenadier having a higher likelihood of detection (B = 0.738) than blacksmelt (*B* = 0.603). Both species were associated with depths between 800 and 1600 m (figure 4*b*).

The indicator species analysis was repeated with the *P. ventilabrum* subset (*N* = 23) of the Northeast Atlantic data to identify indicator species of MPA sites. Four species were significant indicators of MPA sites (figure 4*c*). These species included the moray wolf eel (*Lycenchelys muraena*), Atlantic eelpout (*Lycodes terraenovae*), Arctic telescope (*Protomyctophum arcticum*) and Vahl's eelpout (*Lycodes vahlii*), all of which had high specificity (A = 0.999, 0.999, 1.0 and 1.0, respectively) to MPAs. The moray wolf eel and the Atlantic eelpout both shared the highest association with MPAs (stat(s) = 0.756, *p* < 0.05) (electronic supplementary material, table S6).

## 3. Discussion

The retrieval of fish sequences from sponge specimens previously collected for other monitoring purposes provides perhaps the most attractive demonstration to date of the role of sponges as practical, cost-effective, universal natural DNA samplers for aquatic biodiversity studies. We confidently detected at least 65 teleost and elasmobranch species that could be used to distinguish fish assemblages and identify indicator species associated with depth and biogeographic regions within the North Atlantic.

Congruent with what we know about sponge nsDNA *ex situ* [12], some sponge species appeared to perform better than others. The original experimental design considered 93 sponge specimens; however, only 64 of them were selected for sequencing because they showed amplification of the desired target DNA region (i.e. bands on agarose gels). After bioinformatic quality control, DNA information from 54 individual sponges was retained. Of the 34 *G. hentscheli* samples attempted, only 17 were sequenced and nine were kept after rarefaction. Sample loss occurred, although to a lesser degree, also for *G. barretti* (i.e. 33 attempted, 21 sequenced, 19 kept). *P. ventilabrum* resulted instead in a 100% success rate (*n* = 26), followed by *G. barretti* (58%) and *G. hentscheli* (26%). Curiously, *P. ventilabrum* likely has higher pumping rates and lower microbial abundance than the *Geodia* species [15,16]. It is possible that higher microbial abundance could contribute to increased rates of eDNA decay within sponges due to decomposition by bacteria [15] and less need to derive energy from the uptake of dissolved organic carbon [17]. Given these observed coincidences, the relationships between sponge physiology and nsDNA efficacy deserve further investigation.

Sponges are part of a growing list of ‘natural sampler’ organisms, from which eDNA analysis is possible. High DNA sampling efficiency in some sponge species (i.e. *P. ventilabrum*) is an obvious advantage for biomonitoring, yet the percent success rate of the tetractinellid (*Geodia*) sponges was comparable to or even better than other organisms that have been tested as natural DNA samplers. For example, various leech species have been used to detect prey DNA, with vertebrate detection rates ranging from 9% to 80% of attempted specimens [18]. Similarly, when gut contents of the European brown shrimp (*Crangon crangon*), a generalist scavenger, were analysed with DNA metabarcoding to reconstruct estuarine fish assemblages [19], up to eight stomachs had to be pooled, per DNA extraction, to constitute a sufficient sample. Extraction pooling could represent an appropriate methodological solution for favourable and widespread sponge species with moderate amplification success, such as *G. hentscheli* (i.e. 26%).

The detected fish communities significantly differed among biogeographic regions of the North Atlantic (figure 2*a*), and depth was identified as the most important variable in shaping β-diversity (electronic supplementary material, table S4). Several fish species seemed to be more associated with either the west or east North Atlantic. Thickback sole (*Microchirus virens*) was unique to the Northeast Atlantic; saithe (*Pollachius virens*) and Norway pout (*Trisopterus esmarkii*) were present in the east Atlantic far more than the North American Boreal. Seven species, most of commercial value, were identified as significant indicators of both region and depth. Fishes known to be deep-sea adapted were indeed significantly associated with greater depths, for instance, Rakery beaconlamp (*Lampanyctus macdonaldi*) from 800–1600 m and small-eyed rabbitfish (*Hydrolagus affinis*) from 1200–1600 m. Moreover, the mesopelagic silvery lightfish (*Maurolicus muelleri*) was significantly associated with all sampled depth layers, except for 200–500 m, suggesting that the nsDNA signal detected their flexible migratory behaviour [20]. Interestingly, Atlantic cod (*Gadus morhua*) was associated with the same region and depth range as the silvery pout (*Gadiculus argenteus*), which could be indicative of their known predator-prey relationship [21].

Fish assemblages under different MPA status were distinguishable within the subset of *P. ventilabrum* specimens from the Northeast Atlantic, and greater species richness was observed in specimens from MPAs (figure 2*c,d*). Indicator species associated with MPAs were mostly benthic, such as the moray wolf eel (*Lycenchelys muraena*), which preys on crustaceans and other invertebrates that take refuge in sponge grounds [22]. Atlantic eelpout (*Lycodes terraenovae*) and Vahl's eelpout (*Lycodes vahlii*) were also indicators and known to eat sponge remains and cryptofaunal organisms such as brittle stars [23]. Notably, with traditional survey methods *Lycodes* sp. have been found to correlate positively with high sponge biomass [24]. Differences between the sponge aggregations were strong (*R*^2^ = 0.52) though when pairwise comparisons were made, only one pair, the Faroe Shetland Sponge Belt and Rockall Bank, was identified as a potential driver of the difference. While the significant difference between MPA status was modest (*R*^2^ = 0.09), with adequate samples sizes and targeted rather than opportunistic sampling, sponge nsDNA shows promise for more fine-scale biodiversity surveying.

Environmental DNA analysis is an emerging tool for deep-sea biodiversity [25–27] and ecological studies [28–30], yet eDNA is less abundant in the deep-sea, such that larger volumes of water are needed to attain representative samples, and the manual labour required to filter those samples *in situ* can become a limitation [31]. Furthermore, remote, deep-sea habitats are expensive to reach in the first place, so leveraging of natural samplers in this context represents a major boost for large scale ocean exploration and monitoring. For instance, the specimens in this study had previously been used to understand sponge phylogenetics and connectivity of deep-sea environments [32,33].

The deep sea and high seas are subject to threats such as overfishing [34], deep-sea mining [35], climate change and pollution [36]; sponges are habitat-forming organisms [37] that provide shelter for cryptic animals, thereby also attracting larger more mobile predators [38], and as such play a fundamental role in the structure and functioning of marine ecosystems. Now, the wealth of environmental, biological and molecular data that can be comprehensively obtained from sponges significantly expands their broader value in marine ecology and conservation.

## 4. Methods

### (a) Specimen selection

Three sponge species—*Phakellia ventilabrum* (*n* = 26, order Bubarida), *Geodia barretti* (*n* = 21, order Tetractinellida) and *Geodia hentscheli* (*n* = 17, order Tetractinellida)—from various North Atlantic sponge grounds were selected for sequencing (*n* = 64 of which 54 were analysed for the study—see statistical analysis below), all collected previously for the SponGES project (www.deepseasponges.org), which ran until 2020 (electronic supplementary material, table S1). The sponges were stored in 100% EtOH which was replaced at least once to maintain a high percentage of EtOH, since the water retained by the sponges can significantly dilute the preservative. For each DNA extraction, between 1–1.5 cm^3^ of sponge tissue was used. For the *Geodia* spp., we avoided the cortex (with less cells than the choanosome), but for *Phakellia ventilabrum* we used pieces containing both the pinacoderm and choanosome; these decisions were made based on the original purpose of these specimens which was for phylogenetic analysis but recent research has shown that for the purpose of eDNA metabarcoding analysis the particular part of the sponge biopsied does not significantly change the results [39]. The sponge DNA had been extracted between 6 and 36 months after sampling using the Qiagen DNeasy Blood and Tissue Kit (Hilden, Germany), optimal for sponge nsDNA extraction [39] and were stored at the Natural History Museum, London, at −80°C until being transported to −20°C freezers at Liverpool John Moores University.

### (b) Library preparation and sequencing

DNA extracts were diluted with molecular grade water to between 30 and 50 ng µl^−1^. DNA was amplified using PCR with the Tele02 primers [14]. The forward sequence Tele02-F (5′-AAACTCGTGCCAGCCACC-3′) and the reverse sequence Tele02-R (3′-GGGTATCTAATCCCAGTTTG-5′), were used to target a 167 bp fragment of the mitochondrial 12S rRNA gene. PCRs were prepared to a total volume of 20 µl for each sample and included 10 µl of 2X MyFi Mix (Meridian Bioscience), 1 µl of each forward and reverse primer, 0.16 µl Bovine Serum Albumin (Thermo Fisher Scientific), 5.84 µl molecular grade water, and 2 µl of diluted DNA extract. The samples were amplified in triplicate across two libraries using the following conditions: 95°C for 10 min, followed by 35 cycles of 95°C for 30 s, 60°C for 45 s, 72°C for 30 s, and finishing at 72°C for 5 min followed by a 4°C hold. Negative controls (*n* = 5) and positive controls (*n* = 5), which were molecular grade water and a single fish species not present in the North Atlantic (iridescent catfish *Pangasionodon hypopthalmus*) respectively, underwent PCR alongside the samples. PCR triplicates were pooled and visualized on a 2% agarose gel (150 ml 1X TBE buffer with 3 g agarose powder) stained with 1.5 µl SYBRsafe dye. PCR products were individually purified using a double-size selection in 1 : 1 and 0.6 : 1 ratio of Mag-Bind Total Pure NGS magnetic beads (Omega Bio-Tek) to PCR product. Products were visualized on an agarose gel again to assure purity (i.e. target length bands on agarose gels were visible with minimal to no other bands present). Purified PCR products were quantified using a Qubit dsDNA HS Assay kit (Invitrogen), and pooled equimolar into their corresponding libraries (i.e. pooled samples each contained unique 8-bp dual barcodes). Pooled libraries were imaged on a Tape Station 4200 (Agilent) to check the purity of the libraries. The libraries were then purified based on the Tape Station results, double-size selecting the target fragment using magnetic beads as explained before. A unique adapter sequence was ligated to each library using the NEXTFLEX Rapid DNA-Seq Kit for Illumina (PerkinElmer) following the manufacturer protocol. After adapter ligation, the libraries were again imaged on the Tape Station and purified with magnetic beads, this time with a 0.8 : 1 ratio of beads to sample, as per the NEXTFLEX Rapid DNA-Seq Kit instructions. The dual-indexed libraries were then quantified by qPCR using the NEBNext Library Quant Kit for Illumina (New England Biolabs). The libraries were pooled at equimolar concentrations having a final molarity of 50 pM with a 10% PhiX spike-in. The libraries were sequenced at Liverpool John Moores University on an Illumina iSeq100 using iSeq i1 Reagent v2 (300 cycles).

### (c) Bioinformatics pipeline

The sequences were quality controlled through the following series of steps using Python v2 within the OBITOOLS 1.2.11 [40] package. The raw sequences were trimmed to a length of 150 bp using the command ‘obicut’ to remove low-quality bases from the ends which were determined from the output of the ‘fastqc’ command. The trimmed reads were then merged using ‘illuminapairedend’, from which any paired-end alignments with low (less than 40) quality scores were removed. The remaining paired-end alignments were demultiplexed using ‘ngsfilter’, filtered by length (130–190 bp) and dereplicated using ‘obiuniq’. Chimeras were removed de novo using the programme VSEARCH version 2.4.3 [41]. The remaining sequences were then clustered using the programme SWARM v2 [42] with ‘*d*-value’ = 3. Taxonomy was assigned using the Bayesian LCA-based taxonomic classification method (BLCA) [43]. We first created a database using ‘ecoPCR’ from OBITOOLS with the Tele02 primers against the EMBL database (release version r143). This database was combined with a trained BLCA custom database containing fish species, specifically Teleosts and Elasmobranchs, (custom database file can be found here: https://doi.org/10.5061/dryad.rbnzs7hhp [44]). The workflow of BLCA was followed and can be found at: https://github.com/qunfengdong/BLCA. This resulted in taxonomic assignments where each level (i.e. family, genus) was associated with a percent probability of correct assignment. Analyses were carried out with taxonomies that had *a* ≥ 99% probability of correct assignment to reference sequences (i.e. species referenced in this study had *a* ≥ 99% identity at the species level and 100% identity at all higher levels of assignment to reference sequences).

### (d) Statistical analysis

All downstream analyses were done using R version 4.1.3 [45]. The MOTUs were decontaminated by removing the highest number of reads of a contaminant present in either the PCR positive control or PCR negative control from all samples (electronic supplementary material, figure S3). Ten samples that had less than 100 reads were removed from the dataset based on a rarefaction curve (read counts) suggesting species saturation after 100 reads (electronic supplementary material, figure S1). Using the R package vegan v 2.5.7 [46], β-diversity was examined through multi-dimensional scaling of a Jaccard dissimilarity matrix (presence–absence) of teleosts and elasmobranchs detected from each sponge, comprising of only MOTUs identified to the species level. We tested the homogeneity among the group dispersions of biogeographic regions and sponge species using the functions ‘betadisper‘ and ‘anova‘, then tested for significant differences in β-diversity between regions, sponge species and region and sponge species as interacting terms, by permutational multivariate analysis of variance (PERMANOVA) using the function ‘adonis’. The same tests (excluding sponge species as an explanatory variable) were repeated for all *G.barretti* samples (electronic supplementary material, figure S4, electronic supplementary material, table S7) and for the *P. ventilabrum* subset of the Northeast Atlantic. Pairwise comparisons of the biogeographic groups and population groups were performed, and *p*-values were corrected with the Benjamini-Hochberg method [47]. Correlations of fish assemblages with latitude, sampling depth and sampling year were tested using the function ‘envfit‘. All tests on β-diversity were done on Jaccard dissimilarity matrices and underwent 1000 permutations. The ‘accumcomp‘ function from the BiodiversityR package v 2.14.2.1 [48] was used to create species accumulation curves. Using the R package indicspecies v 1.7.12 [49], an indicator value species analysis and multilevel pattern analysis was done using the function ‘multipatt‘ with IndVal.g method on the same Jaccard dissimilarity matrix of species for sampling depth ranges, biogeographic regions and MPA status in the Northeast Atlantic with *P. ventilabrum* samples. Tests underwent 10 000 permutations. All figures were generated using the R packages tidyverse v 1.3.1 and ggplot2 v 3.4.0 [50,51]. All raw data and code can be found through the links in the data accessibility statement.

## Data Availability

The raw sequencing data files can be accessed at https://doi.org/10.5281/zenodo.7740858 [52]. The code and other data can be accessed at https://doi.org/10.5061/dryad.rbnzs7hhp [44]. Additional information is provided in the electronic supplementary material [53].
